# The confounded crude case-fatality rates (CFR) for COVID-19 hide more than they reveal—a comparison of age-specific and age-adjusted CFRs between seven countries

**DOI:** 10.1371/journal.pone.0241031

**Published:** 2020-10-21

**Authors:** Manfred S. Green, Victoria Peer, Naama Schwartz, Dorit Nitzan

**Affiliations:** 1 School of Public Health, University of Haifa, Haifa, Israel; 2 World Health Organization, European Region, Copenhagen, Denmark; National Institute for Infectious Diseases Lazzaro Spallanzani-IRCCS, ITALY

## Abstract

**Background:**

Crude case-fatality rates (CFRs) for COVID-19 vary widely between countries. There are serious limitations in the CFRs when making comparisons. We examined how the age distribution of the cases is responsible for the COVID-19 CFR differences between countries.

**Methods:**

COVID-19 cases and deaths, by ten-year age-groups, were available from the reports of seven countries. The overall and age-specific CFRs were computed for each country. The age-adjusted CFRs were computed by the direct method, using the combined number of cases in all seven countries in each age group as the standard population. A meta-analytic approach was used to obtain pooled age-specific CFRs.

**Findings:**

The crude overall CFRs varied between 0.82% and 14.2% in the seven countries and the variation in the age-specific CFRs were much smaller. There was wide variation in the age distribution of the cases between countries. The ratio of the crude CFR for the country with the highest CFR to that with the lowest (6.28) was much lower for the age-adjusted CFRs rates (2.57).

**Conclusions:**

The age structure of the cases explains much of differences in the crude CFRs between countries and adjusting for age substantially reduces this variation. Other factors such as the definition of cases, coding of deaths and the standard of healthcare are likely to account for much of the residual variation. It is misleading to compare the crude COVID-19 CFRs between countries and should be avoided. At the very least, age-specific and age-adjusted CFRs should be used for comparisons.

## Background

Human infections caused by the novel coronavirus SARS-2-CoV 2019 were first identified in Wuhan in China in early December 2019. On January 30, 2020, the World Health Organization (WHO) reported the spread of the disease a Public Health Emergency of International Concern (PHEIC) [1]. On March 11, 2020, WHO declared COVID-19 as a pandemic. By October, 2020, almost all countries had been affected, and globally there were reports of more than 37 million cases and more than a million deaths. Early estimates indicated that the average proportion of deaths among the diagnosed cases, defined as the case-fatality rate (CFR), was around 2.3% [2]. However, subsequently, the reported crude COVID-19 CFRs varied widely between countries [3, 4]. The limitations of comparing crude CFRs in general, has been evaluated previously [5]. The strong positive association between the COVID-19 CFR and age has been demonstrated both in observational studies [6] and in a model-based analysis [7], particularly over the age of 40.

The interpretation of the CFR depends on the context in which it is used. In a single cohort of patients, it can indicate the severity of the disease at a single point in time. It can also be used to assess trends in the impact of changes in health care over time. In the current context of the COVID-19 pandemic, CFRs are commonly compared between countries and that may lead to speculation on differences in healthcare. For example, it may be concluded that the CFRs somehow reflect the successes and failures of the different countries in the treatment of serious cases. In general a number of factors could impact on the both the numerator and denominator of the CFR. This is especially important for the CFR for COVID-19. There could be misclassification of causes of death and there are likely to be variations in the definition of cases in the denominator. Substantial confounding by age could impact on CFR comparisons between different groups. A related concept is the infection fatality rate (IFR) which includes asymptomatic cases in the denominator, which need to be identified by screening tests. Since the IFR is rarely available for COVID-19, in this paper we consider only the CFR. In this paper, we examined the contribution of the age distribution of the cases when comparing the COVID-19 CFRs between seven countries, with widely varying CFRs.

## Methods

We studied published crude and age-specific CFRs in cohorts of cases of COVID-19 in seven countries, with varying periods of follow-up. The countries chosen were based on the accessibility of the data. The first case COVID-19 in Israel was confirmed on 21 February 2020. The first case in South Korea was announced on 20 January 2020.The COVID-19 spread from Hubei Province, China, after December 2019.SARS-CoV-2 was confirmed to have reached Spain, Sweden, Italy, and Canada on end of January 2020. The data on cases and deaths by age group were available from China on February 11, 2020. For South Korea cases and deaths were updated to the end of April. For Spain, the cases and deaths were updated to mid-May 2020.The information for Israel, Italy, Canada and Sweden were updated during August 2020. The data for each country were not necessarily updated to the time of the study, and the CFR’s may have changed over time. Age-specific data on the cases and deaths by ten-year age groups (0–9, 10–19, …..80+), were available for seven countries: Italy [8], Spain [9], Sweden [10], China [11], S Korea [12], Israel (Ministry of Health, personal communication) and Canada [13]. The outcome variable was defined as the crude CFR defined as the number of deaths divided by the number of reported cases. The exposure variable was the individual country. Age-group was considered as a confounding variable. Age-adjustment was carried by the direct method, using the distribution of the combined cases of all six countries by age group as the standard population. 95% confidence intervals were computed for each age-adjusted rate using WinPepi [Version 11.65, Aug, 2016].

### Ethical considerations

Open access aggregative and anonymous data were used and there was no need for ethics committee approval.

## Results

The age-specific number of cases, number of deaths and the crude CFRs by country are given in Table 1. The distributions of the cases vary markedly between the countries. For example, Israel and South Korea are heavily weighted in the 20–39 age group, China has a more balanced distribution and Italy, Sweden and Canada are heavily weighted in the over 70 age groups.

**Table 1:** Deaths/cases of COVID-19 and CFRs (%) by age group, for seven countries.

Country	Age group (years)								
	0–9	10–19	20–29	30–39	40–49	50–59	60–69	70–79	80+
**China**	0/416	1/549	7/3619	18/7600	38/8571	130/10008	309/8583	312/3918	208/1408
** CFR (%)**	**0.00**	**0.18**	**0.19**	**0.24**	**0.44**	**1.30**	**3.60**	**7.96**	**14.77**
**Italy**	4/2784	0/4964	16/15991	67/20847	313/33082	1233/44392	3582/32887	9285/34788	21144/61297
** CFR (%)**	**0.14**	**0.00**	**0.10**	**0.32**	**0.95**	**2.78**	**10.89**	**26.69**	**34.49**
**S. Korea**	0/141	0/586	0/2940	2/1147	3/1421	15/1953	35/1347	72/708	115/485
** CFR (%)**	**0.00**	**0.00**	**0.00**	**0.17**	**0.21**	**0.77**	**2.60**	**10.17**	**23.71**
**Spain**	2/877	5/1637	23/13461	63/22639	201/35135	611/42794	1695/34360	4632/32443	11954/55779
** CFR (%)**	**0.23**	**0.31**	**0.17**	**0.28**	**0.57**	**1.43**	**4.93**	**14.28**	**21.43**
**Israel**	0/10966	2/19661	2/21722	3/14722	10/13201	37/10343	77/6900	195/3436	526/2503
** CFR (%)**	**0.00**	**0.01**	**0.01**	**0.02**	**0.08**	**0.36**	**1.12**	**5.68**	**21.01**
**Sweden**	1/595	0/3883	10/14062	16/13694	45/14418	162/15403	401/8728	1252/6046	3930/10225
** CFR (%)**	**0.17**	**0.00**	**0.07**	**0.12**	**0.31**	**1.05**	**4.59**	**20.71**	**38.44**
**Canada**	no data	no data	9/18885	15/17658	50/18115	210/17872	650/11564	1629/8395	6404/18669
** CFR (%)**	**no data**	**no data**	**0.05**	**0.08**	**0.28**	**1.18**	**5.62**	**19.40**	**34.30**

**Fig 1:**
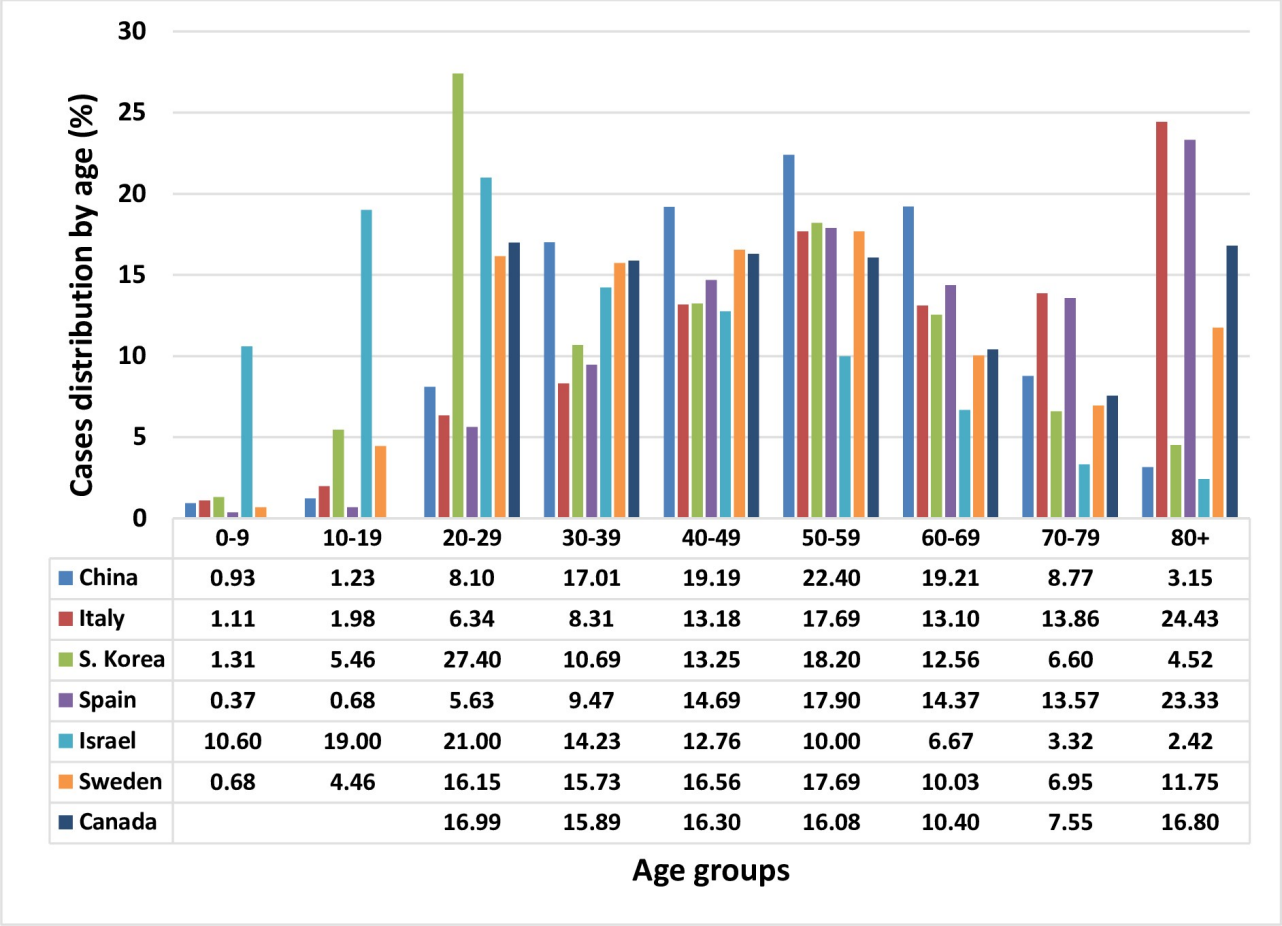
Distribution of cases (%) by age for seven countries–China, Italy, S Korea, Spain, Israel, Sweden, and Canada.

The distributions of the cases for each country are shown in Fig 1. It is clear that distributions vary widely and are not necessarily related to the age distribution of the population of the country. For example, for South Korea, there is a relatively large number of cases in the age group 20–29, due to an outbreak affecting that age group in particular.

**Fig 2:**
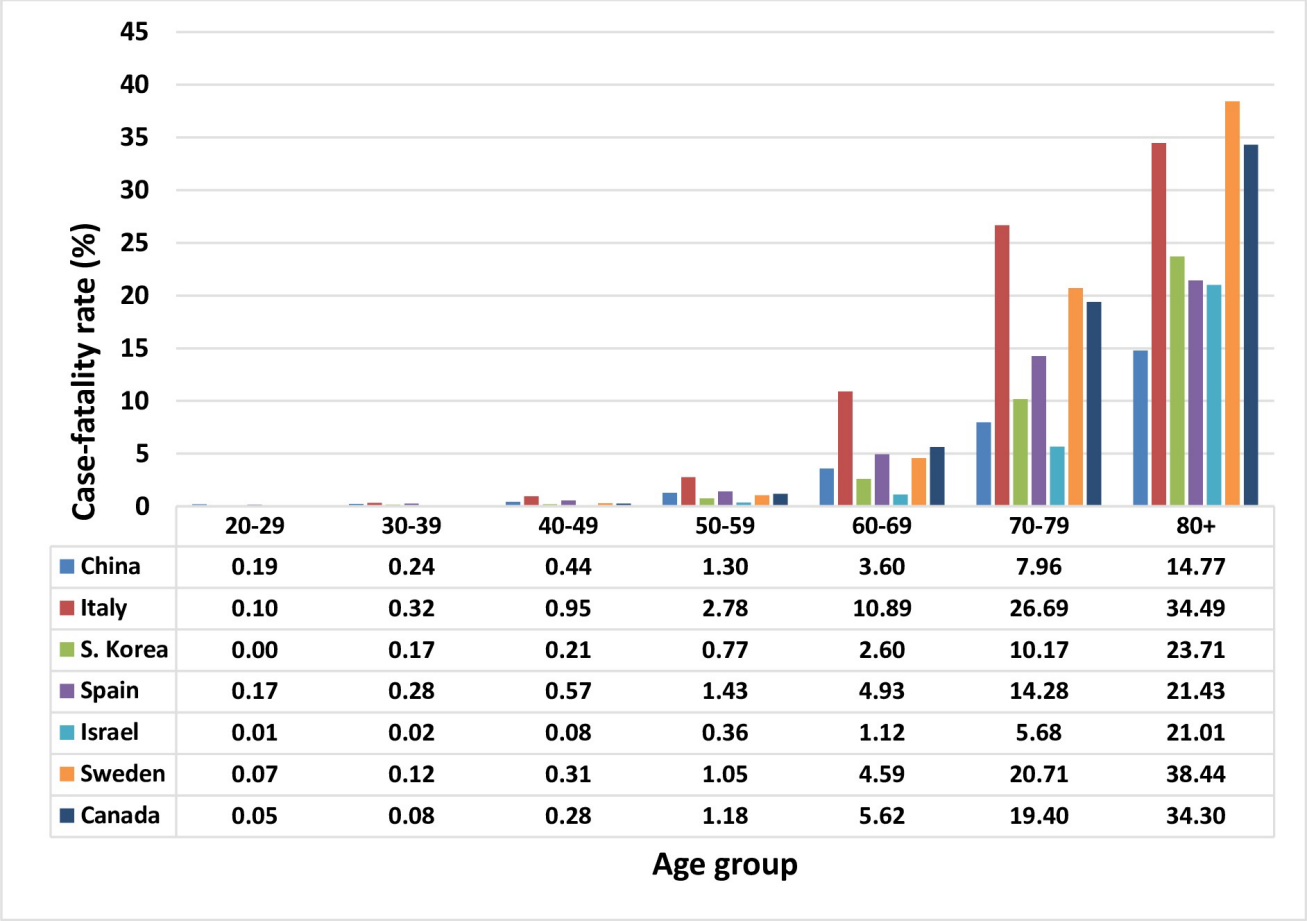
Age specific case-fatality rates (%) for seven countries–China, Italy, S Korea, Spain, Israel, Sweden, and Canada.

The age-specific CFRs are shown in Fig 2. While there are differences in the age-specific CFRs between countries, the trend of steeply increasing CFRs in the oldest age groups is evident. Age groups 0–9 and 10–19 were excluded from the figure, since there were almost no deaths.

The crude (%) and age-adjusted CFRs (%) are compared in Table 2. The crude CFRs varied from 0.82% for Israel to 14.20% for Italy and the ratios of the crude CFRs compared with lowest CFR, varied between 0.36 and 6.28. The age-adjusted CFRs varied between 4.20% for China to 10.80% for Italy and the ratios of the age-adjusted CFRs for each country compared with the lowest CFR adjusted varied between 1.00 and 2.57.

**Table 2:** Crude and age standardized (95%CI) case-fatality rates (CFR,%) for COVID-19 in seven countries.

Country	Crude CFR (%)	Age-adjusted CFR rate (%)	95% CI	Ratio to lowest crude CFR	Ratio to lowest age-adjusted CFR
China	2.29	4.20	3.91–4.54	1.01	1.00
Israel	0.82	4.49	4.20–4.78	0.36	1.07
S. Korea	2.26	5.72	5.00–6.43	1.00	1.36
Spain	8.02	6.24	6.16–6.32	3.54	1.49
Canada	8.07	8.98	8.82–9.19	3.57	2.14
Sweden	6.68	9.71	9.50–9.92	2.96	2.31
Italy	14.20	10.80	10.72–10.92	6.28	2.57

**Fig 3:**
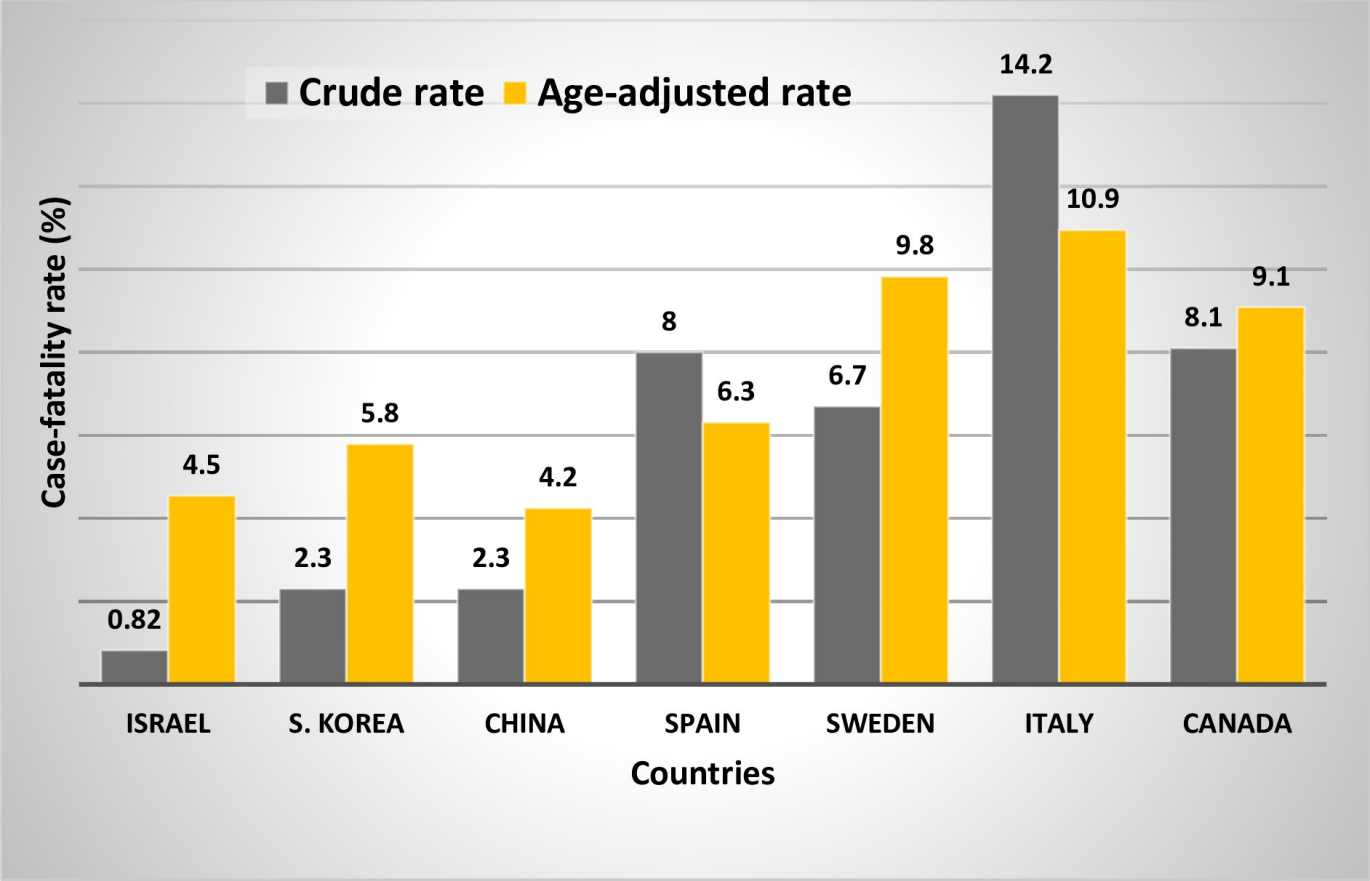
Crude (%) and age-adjusted case-fatality rates (%) by country for seven countries.

Fig 3 shows graphically the marked reduction in the differences between the crude (%) and age-adjusted CFRs (%) for the seven countries.

### Meta-analyses by age group

**Fig 4:**
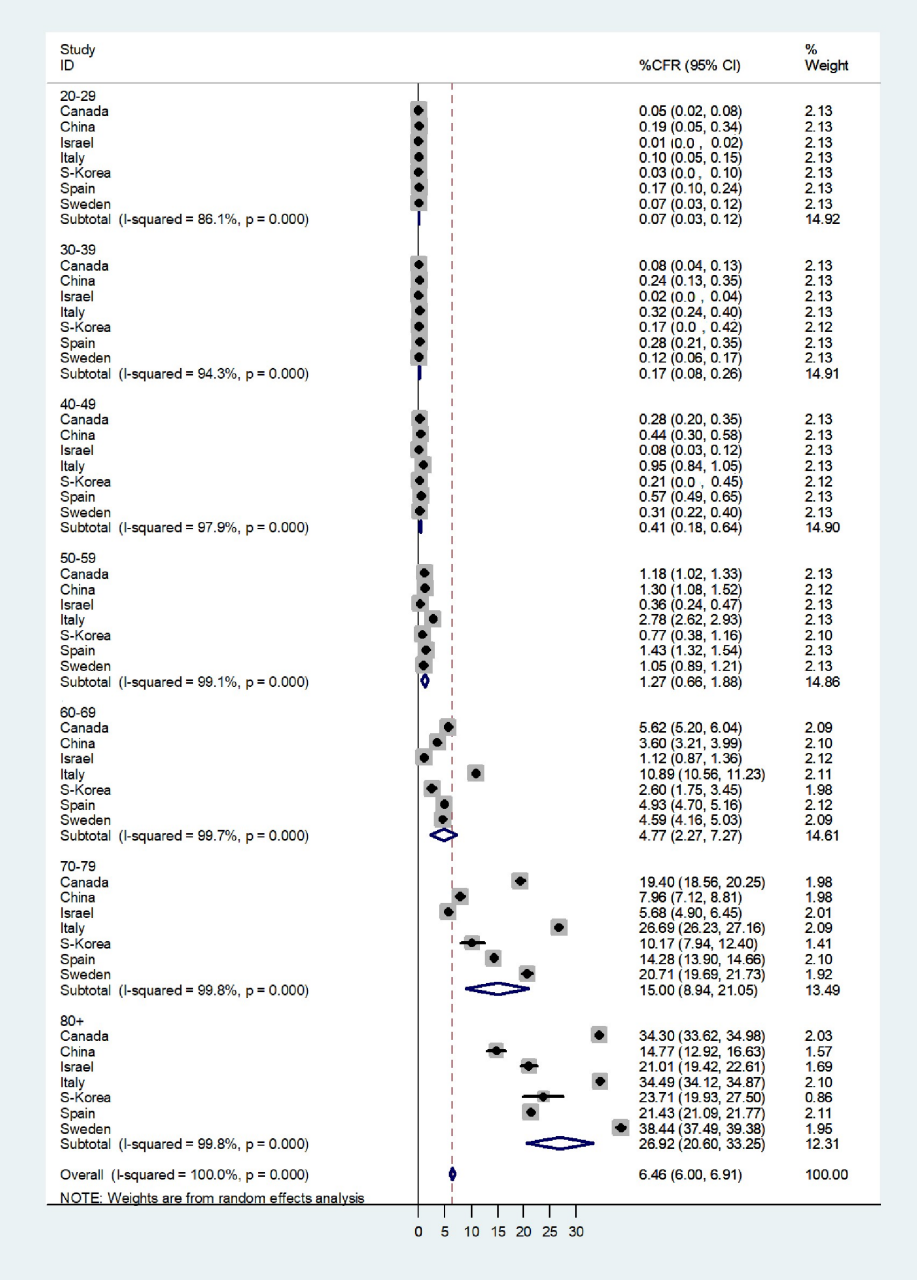
Forest plot of case-fatality rates (CFR %) by age group and country.

We used meta-analytic methods to obtain weighted pooled estimates of the CFR’s by age group. The results of the meta-analysis are presented in the forest plot in Fig 4, which shows the CFRs by age group and country and the pooled CFR’s for each age group (results are shown start at age 20, since there so few deaths in the age group under 20). There was considerable variation in the CFR’s by country within each age group, but the trends by age were very similar, increasing steeply after age 60. The pooled CFR’s were 0.07% in the age group 20–29, 0.17% for 30–39, 0.41% for 40–49, 1.27 for 50–59, 4.77% for 60–69, 15% for 70–79 and 26.92% for 80+. The meta-regression showed that the age groups contributed most of the differences in the CFRs (p<0.001).

## Discussion

In our study, we examined crude, age-specific and age-adjusted CFRs for COVID-19 in seven countries, with widely varying crude CFRs. The trends in the age-specific CFRs were remarkably similar in the seven countries, with the CFR’s increasing steeply in those over 70. After adjusting for age, the marked differences in the crude CFRs were substantially reduced. These findings demonstrate the importance of accounting for age when comparing rates in general and CFRs in particular. The results of this study are strengthened by the use of national data or large datasets from a number of countries, with considerable differences in the extent of the pandemic in each country. It should be stressed that the age distribution of the cases was used to compute the age-adjusted CFRs and not the age distribution of the total population in each country.

In addition to the age distribution of the cases, the use of the CFR for comparisons between countries has other important limitations. Selection bias is clearly present when calculating the denominator on the basis of reported cases. As mentioned, the CFR must be distinguished from the IFR, which includes asymptomatic cases identified by deliberate or incidental screening with diagnostic tests. In addition, if only those with more severe symptoms are tested this will affect the denominator of the CFR and will depend on the testing strategy of each country. If more mild cases are identified, this is likely to reduce the CFR. there is a lag time between the reporting of the case and the death which can occur up to weeks later. In the country reports, cases and deaths are usually reported at the same time, so the cases in the denominator are usually an overestimate of the true denominator which should be the number of cases reported sometime earlier [14, 15]. This will have a more dramatic effect when the number of cases are rising rapidly. Selection bias may also affect the numerator if only deaths occurring in hospital are reported. Information bias can be present in both the numerator and denominator of the CFR. The definition of the cases may be biased due to the variability of the sensitivity and specificity of the diagnostic tests for COVID-19. Information bias in the numerator can occur when the cause of death is coded. This could be particularly problematic in elderly people with multiple co-morbidities.

The purpose of this paper is to demonstrate the dramatic effect of confounding by the age distribution of the cases when using crude overall CFRs for country comparisons. This was shown in an earlier paper when comparing six countries, and we have extended it to a comparison of seven countries with widely different CFRs. The age structures of the population of the seven countries used in this study vary markedly. The percentage of the population age 65 and over is 12% in Israel, 23% in Italy 23%, 9.3% in S Korea, 19.6% in Spain, 20% in Sweden, 11% in China and 17.6% in Canada [16]. However, the main impact of confounding by age was due to the differences in the age distribution of the cases. This was largely due to the specific circumstances of exposure. For example, most of the cases in Italy occurred in an area of a particularly old population [3]. In some countries, many of the cases were medical personnel, a large number of whom were relatively young women [17]. In South Korea, a large percentage of the cases were young women associated with a specific religious group [18]. In Germany, many of the cases were relatively young people returning from skiing holidays in Austria and Italy [19]. In Israel, the largest outbreaks occurred in the ultra-orthodox Jewish community, where the number of children per family is much higher than in the general population. Other factors affecting the age distribution of the cases, depended on the frequency of outbreaks in homes for the elderly [20].

The results of this study once again demonstrate the pitfalls of comparing unadjusted rates. The assumption that differences between countries in testing policies or standard of treatment accounted for the wide discrepancies in CFRs, is not well-founded. This does not mean that there are no differences. For example, it is possible that where the health services were overloaded, younger patients were more likely to be admitted to intensive care units with better chances of survival. Clearly, the data are incomplete and other factors affecting CFRs such as case definitions, use of different denominators, underlying health conditions and the standard of health services are likely to play important roles. In order to assess the impact of these factors, age-specific and age-adjusted CFRs must be used.

## Conclusions

In addition to the selection and information biases inherent in computing CFRs, the age structure of the cases dramatically impacts on the differences in the crude CFRs between countries. Failure to account for this source of confounding markedly distorts the country comparisons. The substantial reduction in the differences in the age-adjusted CFRs suggest that differences in the standard of healthcare between these countries may not play as important a role in affecting the death rates, as some have hypothesized. Crude COVID-19 CFRs have no real use for between country comparisons and should be avoided. In general, for comparisons between groups and countries, age-adjusted CFRs can be used, but age-specific COVID-19 CFRs are generally far more meaningful.
